# Mining novel cell glycolysis related gene markers that can predict the survival of colon adenocarcinoma patients

**DOI:** 10.1042/BSR20201427

**Published:** 2020-08-13

**Authors:** Sihan Chen, Guodong Cao, Wei Wu, Yida Lu, Xiaobo He, Lei Yang, Ke Chen, Bo Chen, MaoMing Xiong

**Affiliations:** Department of General Surgery, The First Affiliated Hospital of Anhui Medical University, Hefei, Anhui China

**Keywords:** Colon adenocarcinoma, Glycolysis, mRNA, Prognostic, Survival, tumor microenvironmentsts

## Abstract

Colon adenocarcinoma (COAD) is a malignant gastrointestinal tumor, often occurring in the left colon, which is regulated by glycolysis-related processes. In past studies, multiple genes that influence the prognosis for survival have been discovered through bioinformatics analysis. However, the prediction of disease prognosis using a single gene is not an accurate method. In the present study, a mechanistic model was established to achieve better prediction for the prognosis of COAD. COAD-related data downloaded from The Cancer Genome Atlas (TCGA) were correlated with the glycolysis process using gene set enrichment analysis (GSEA) to determine the glycolysis-related genes that regulate COAD. Using COX regression analysis, glycolysis-related genes associated with the prognosis of COAD were identified, and the genes screened to establish a predictive model. The risk scores of this model were correlated with relevant clinical data to obtain a connection diagram between the model and survival rate, tumor characteristic data, etc. Finally, genes in the model were correlated with cells in the tumor microenvironment, finding that they affected specific immune cells in the model. Seven genes related to glycolysis were identified (PPARGC1A, DLAT, 6PC2, P4HA1, STC2, ANKZF1, and GPC1), which affect the prognosis of patients with COAD and constitute the model for prediction of survival of COAD patients.

## Introduction

Colon adenocarcinoma (COAD) is a form of malignant gastrointestinal tumor that occurs most commonly in the left colon. It is most common in male patients and has the third-highest incidence of all gastrointestinal tumors. According to a WHO 2018 report [1], there are approximately 1.8 million colonic adenocarcinoma patients worldwide, of which 881,000 died of the disease in 2018. Due to the high-fat and low-cellulose foods eaten in parts of China, patients with colon cancer have a high incidence [2–4].

It is currently known that a single gene or molecular marker does not provide a good diagnosis or predict the progression of a disease. Increasing numbers of institutions have adopted multiple genes to build predictive models for disease diagnosis. With the development of high-throughput sequencing technology, an increasing network of public databases have been established whose genetic and clinical data can be exploited to build a prognostic model of related diseases [5].

Tumor microenvironments provide a survival environment for tumor cells, comprising a variety of extracellular matrix proteins and stromal cells that promote the growth and metastasis of tumor cells by a variety of means [6]. The immune microenvironment can utilize glycolysis to promote tumor progression. Yajuan Zhang revealed that tumor-associated macrophages regulate the glycolysis of tumor cells by modulating the phosphorylation of phosphoglycerate kinase PGK1 in tumor cells, thereby promoting the development of pleomorphic glioblastoma [7]. Highly glycolytic tumors exhibit an immunostimulatory tumor microenvironment that bypass immune checkpoints, such as PD-L1, in tumors [8].

Tumors are not controlled by the cell cycle and they promote cellular energy metabolism, as a result of which tumor cells grow and differentiate. Due to the Warburg effect, tumor cells rely on glycolysis for metabolism in the presence of oxygen and produce large quantities of lactic acid [9]. As the tumor adapts to the tumor microenvironment, the tumor cells proliferate more efficiently. Finally, the Warburg effect plays an important role in maintaining the pathway between oxygen-sensitive transcription factors and nutrition-sensitive signals [10]. Using the principal of the one gene-one enzyme hypothesis, additional genes of glycolysis related to the prognosis of COAD were identified and so reveal genes that control the glycolytic process, providing new targets for clinical diagnosis and treatment in the future [10]. Thus, we further explored the relationship between glycolysis and the immune microenvironment to better understand the glycolysis-related gene model constructed in the present study.

Firstly, the association of glycolysis genes with related COAD data downloaded from The Cancer Genome Atlas (TCGA) were explored using gene set enrichment analysis (GSEA), and genes of glycolysis were further screened to identify those that could affect the prognosis of COAD. A risk scores model of glycolysis-related genes was then constructed, in order to predict the clinical prognosis of COAD and explore the mechanisms described by this model. Finally, the relationship between glycolysis risk scores and the immune microenvironment was explored to determine the identity of the relevant immune cells.

Through the analysis of various bioinformatics databases and related tools, we identified seven genes (PPARGC1A, DLAT, 6PC2, P4HA1, STC2, ANKZF1, and GPC1) that affect the prognosis of COAD, allowing a glycolysis risk scores model to be established.

## Methods

### mRNA expression and patient clinical data

mRNA expression data and the clinical data of patients were downloaded from the TCGA Genomic Data Commons (GDC) database (https://portal.gdc.cancer.gov/). The mRNA expression data were recorded as fragments per kilobase of transcript per million mapped reads (FPKM). The clinical data of 452 patients were represented by age, gender, survival time, survival status, and TNM (tumor, lymph nodes, and metastasis) staging [11].

### Definitions of relevant clinical information

T (Stage of primary tumor)T, T1-2: Colon adenocarcinoma had invaded the mucosa or muscle layer, T3-T4: Infiltration of the serosal layer of the colon or other surrounding organs. N (Lymph node metastasis stage), N0: Colon adenocarcinoma has not metastasized into the lymphatic system, N1-3: Varying degrees of lymphatic metastasis. M (Metastasis stage), M0: No distant metastasis, M1: The cancer has undergone distant metastasis. Stage I and II: representing early colon adenocarcinoma. III and IV: advanced colon adenocarcinoma.

### Enrichment analysis

Enrichment analysis was performed on the selected gene sets using GSEA (https://www.gsea-msigdb.org/gsea/login.jsp), and the following pathways that included glycolysis selected, so as to determine the differences between the genes of patients and healthy individuals: BIOCARTA_GLYCOLYSIS_PATHWAY, GO_GLYCOLYTIC_PROCESS, HALLMARK_GLYCOLYSIS, KEGG _ GLYCOLYSIS _GLUCONEOGENESIS, and REACTOME_GLYCOLYSIS. Furthermore, genes for glycolysis were extracted from the analysis performed by GSEA, from which 326 genes were identified. The expression of glycolytic genes in tumor tissue was compared with that in normal tissue using a Wilcoxon test. Where *P*<0.05 and logFC≠0, differences were considered to be statistically significant. Finally, 253 differentially expressed glycolytic genes were identified between tumor and normal tissues.

### Standardization of data processing and construction of risk scores model

The analysis of mRNA was standardized by adopting the Log2 transformation method. Through univariate Cox analysis, genes related to the prognosis of survival were screened out then multivariate Cox analysis was additionally performed to obtain the coefficients associated with overall survival (OS). The mRNA was divided into two groups according to whether or not the Hazard ratio (HR) value was greater than 1, then prognosis was calculated using the formula: ∑ (βn × expression of gene n), where “β” represents the correlation coefficient (coef) of a specific gene. The patients with risk scores in the highest 50% were regarded as the high-risk group, whereas patients with the lowest 50% were considered low-risk.

### Analysis of the relationship between risk scores and survival rate

The survival analysis package available in the R programming environment was used to plot Kaplan–Meier curves (K-M) for risk scores and survival rate. P-values<0.05 were considered statistically significant. The high-risk scores group had a lower survival rate and so was considered clinically meaningful.

### Receiver operating characteristic (ROC) curves for sensitivity and specificity evaluation of the model

The survival ROC package within the R programming environment was used to plot ROC curves. The area under the red curve (AUC) was used to evaluate the characteristics of the model. Values of 0.5–0.7 indicate an acceptable level of accuracy of the model, 0.7–0.9 represents good accuracy, and >0.9 indicates that the accuracy of the model is excellent.

### Plotting of risk curves, survival status charts, volcano maps, and risk heat maps

The R programming language was used to plot the appropriate volcano maps and heat maps of the risk value model using the ggplot2 and pheatmap packages.

### Correlation coefficient plots

The corrplot package of the R programming language was used to generate heatmaps of data correlation coefficients.

### Cox regression analysis forest map and related gene mutation plots

Cox regression analysis forest maps were plotted using the R programming language, where HR values > 1 were considered a risk factor, and *P*-values <0.05 were considered statistically significant. The gene mutation map was derived from data from the cBioPortal database [12].

### Gene expression and survival curve plots

The R programming language was used to generate scatter plots of gene expression and K-M curves of clinical data-related survival rate.

### Network diagrams of biological function enrichment analysis

Cytoscape 3.7.2 software and the ClueGo plugin was used to plot a network diagram of biological function enrichment analysis for glycolysis-related genes from which a risk scores model was constructed [13].

### Statistical analysis

Kaplan–Meier curves were used to evaluate the relationship between the survival data of each variable. Cox regression analysis was used to assess the relationship between the various clinical data and risk scores. *P*-values <0.05 were considered statistically different. The R packages and software described above were used to conduct all relevant statistical analyses and generation of plots.

## Results

### GSEA for the screening of genes

**Figure 1:**
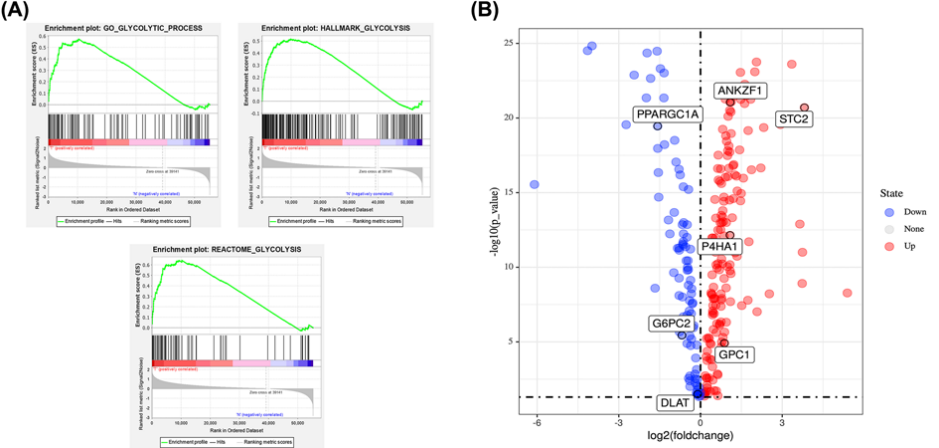
Screen for glycolysis-related genes (**A**) Enrichment plots of three gene sets that were significantly different (*P*<0.05) between normal and COAD tissues by performing GSEA. (**B**) Volcano map of 253 glycolytic genes expressed differentially in tumor and normal tissues (*P*<0.05 and logFc≠0).

A GSEA analysis was performed on the 56753 sets of mRNA expression data from 452 patients downloaded from the TCGA database, and then differences in glycolytic gene expression in COAD compared with normal colon tissue analyzed. Five glycolysis-related gene sets were verified (Table 1), among which GO_GLYCOLYTIC _ PROCESS, HALLMARK_GLYCOLYSIS, and REACTOME _GLYCOLYSIS were significantly expressed (*P*<0.05) (Figure 1A). Using GSEA analysis, the expression of 326 glycolysis-related genes were obtained. The expression of glycolytic genes in colon adenocarcinoma samples compared with normal colon samples was analyzed using a Wilcoxon test. *P*-values <0.05 were considered to represent a statistical difference. Finally, 253 differentially expressed glycolysis genes were identified, of which a volcano map was plotted (Figure 1B).

**Table 1:** Gene sets enriched in COAD (452 samples)

GS<br> follow link to MSigDB	SIZE	ES	NOM *P*-val	RANK AT MAX
BIOCARTA_GLYCOLYSIS_PATHWAY	3	0.520	0.743	10233
GO_GLYCOLYTIC_PROCESS	106	0.572	0.004	10448
HALLMARK_GLYCOLYSIS	200	0.516	0.049	9973
KEGG_GLYCOLYSIS_GLUCONEOGENESIS	62	0.267	0.763	10241
REACTOME_GLYCOLYSIS	72	0.642	0.016	10241

### Screening of glycolytic genes related to the prognosis of survival

A total of 253 glycolysis-related genes were first linked with patient survival data and analyzed using univariate Cox regression analysis to identify genes that affect the survival and prognosis of patients. The results indicated that 11 glycolysis-related genes satisfied this criterion (*P*<0.05), the results of which are displayed in Table 2. Secondly, multivariable Cox regression analysis was used to calculate the expression of 11 genes (ENO3, PPARGC1A, P4HA1, STC2, IDUA, ANKZF1, DLAT, G6PC2, ENO2, PPFIA4, and GPC1), the results demonstrating that 7 (PPARGC1A, P4HA1, STC2, ANKZF1, DLAT, G6PC2, and GPC1) fulfilled those conditions (Table 3), and their risk scores were calculated. HR > 1 was a risk factor, while HR <1 was a protective factor.

**Table 2:** Glycolytic genes related to COAD prognosis

Gene	HR	HR.95L	HR.95H	*P*-value
ENO3	2.18927739	1.36149086	3.5203582	0.00122384
PPARGC1A	0.5975151	0.40841975	0.87416021	0.00798361
P4HA1	1.35025402	1.04160762	1.7503577	0.02333984
STC2	1.2672714	1.05212113	1.52641818	0.0125891
IDUA	1.38298719	1.0054061	1.90236915	0.04624992
ANKZF1	1.89498091	1.18238756	3.03703521	0.00790425
DLAT	0.66790451	0.45209452	0.98673267	0.0426581
G6PC2	9.80E-07	2.14E-12	0.44960029	0.03751194
ENO2	1.3152164	1.06439485	1.62514331	0.01114805
PPFIA4	3.7245012	1.75903092	7.8861088	0.00059143
GPC1	1.39015386	1.10054516	1.75597315	0.00571382

**Table 3:** Genes that constitute glycolysis models that predict COAD prognosis

id	β (coef)	HR
PPARGC1A	-0.3541417	0.7017755
P4HA1	0.47509168	1.60816163
STC2	0.22913696	1.25751426
ANKZF1	0.65451593	1.92421085
DLAT	-0.5339837	0.58626479
G6PC2	-10.47157	2.83E-05
GPC1	0.27174193	1.31224831

### Relationship between risk scores, survival rate, and form of COAD

The risk scores of genes related to glycolysis were obtained using the formula: ∑ (β*n* × expression of gene *n*). The risk scores were allocated into two groups, either high or low, and the difference calculated and plotted on a survival curve. A ROC curve was plotted to judge the reliability of the model and query the relationship between risk scores and disease type. The results indicated that the prognosis of survival in the high-risk group was significantly lower than that of the low-risk group (*P* = 1.348e-11), with an AUC of the ROC = 0.819, demonstrating that the model had good sensitivity.

**Figure 2:**
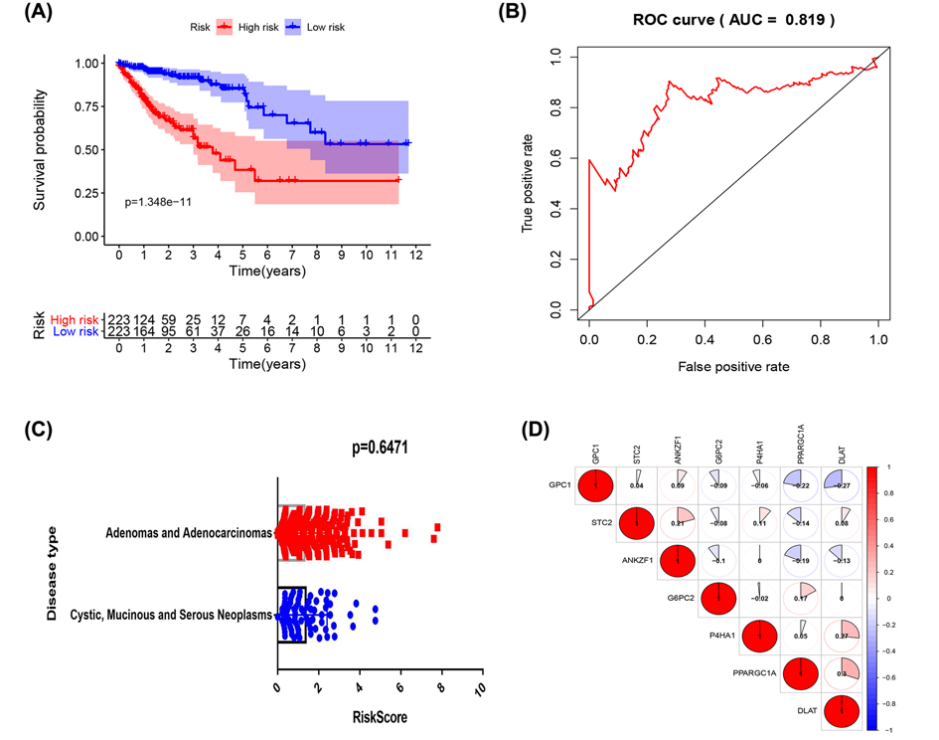
The build of risk scores correlation model (**A**) Analysis of survival prognosis of the constructed glycolysis-related models. Red represents the high-risk group and blue represents the low-risk group. (**B**) Receiver Operating Characteristic (ROC) curve of the glycolysis-related model. (**C**) Differences in risk scores for the two types of colon adenocarcinoma. (**D**) Relationship between the expression of genes of the glycolysis model, red representing a positive correlation, and blue representing a negative correlation. The values represent the degree of correlation.

No significant difference in risk scores was found between the two common disease types of COAD, which confirms that the model in the present study is suitable for the two types of COAD. The co-expression heat map indicates that PPARGC1A has the greatest positive correlation with DLAT. Furthermore, adenomas and adenocarcinomas, and cystic, mucinous and serous neoplasms are the two most common types of diseases in COAD. There was no apparent difference in the risk scores between them, further confirming that the risk scores model can be used for these two types of disease (Figure 2).

### Analysis of seven genes and COAD clinical data for constructing a glycolysis risk model

**Figure 3:**
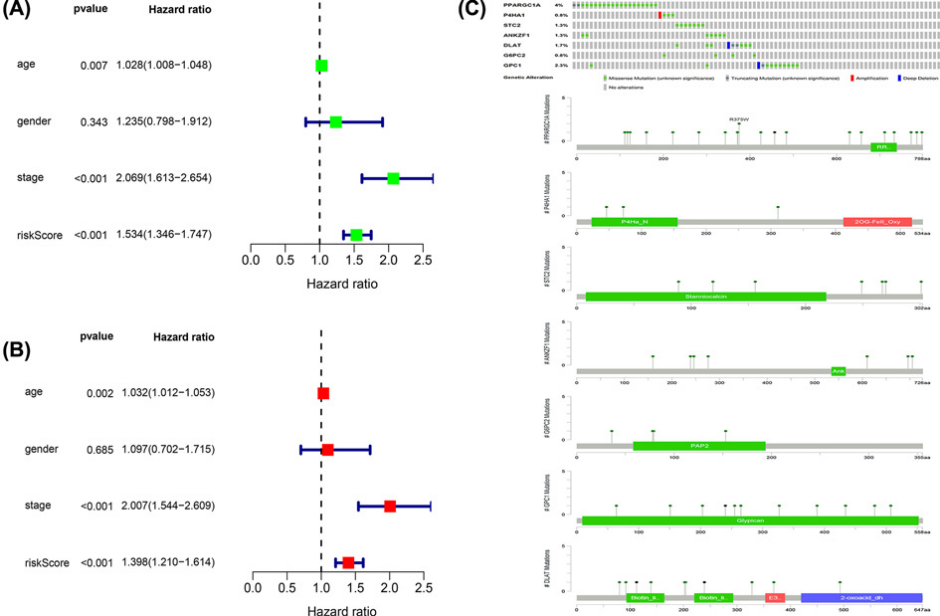
Analysis of risk factors (**A**) Univariate Cox regression analysis of the relationship between glycolysis and related clinical data. (**B**) Multivariate Cox regression analysis of the relationship between glycolysis and related clinical data. (**C**) Gene mutations that constitute the glycolysis model.

From the results of both univariate and multivariate Cox regression analyses, risk scores were found to be an indicator of disease Progression. This establishes that the risk scores were risk factors for COAD with statistical significance (Figures 3A,B).

### Mutation and expression of glycolysis-related genes

**Figure 4:**
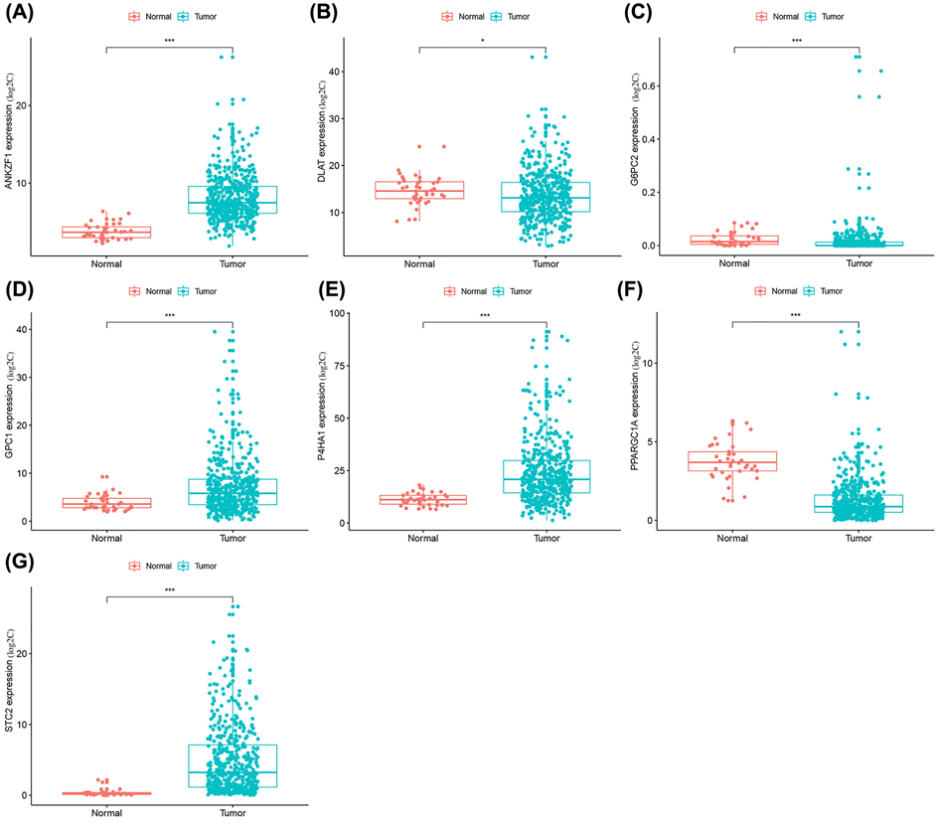
Analysis of mRNAs expression level Expression of 7 mRNAs in colon adenocarcinoma and normal tissues. (**A**) The expression of ANKZF1 in tumors and normal tissue. (**B**) The expression of DLAT in tumors and normal tissue. (**C**) The expression of G6PC2 in tumors and normal tissue. (**D**) The expression of GPC1 in tumors and normal tissue. (**E**) The expression of P4HA1 in tumors and normal tissue. (**F**) The expression of PPARGC1A in tumors and normal tissue. (**G**) The expression of STC2 in tumors and normal tissue (*P<0.05, **P<0.01, ***P<0.001).

The seven genes screened as described in the experiments above were evaluated and analyzed in 452 COAD samples downloaded from the cBioPortal database. The results indicate that 12.2% of genes were mutated. The highest proportion of mutations were in PPARGC1A and involved 7 missense mutation boxes and 2 truncating mutation boxes (Figure 3C). A comparison of the expression levels of the above seven genes in COAD and healthy colon tissue demonstrated that their expression levels were both significantly up-regulated (DLAT, 6PC2, P4HA1, STC2, ANKZF1, and GPC1) and down-regulated (PARGC1A), as displayed in Figure 4.

### Construction of an enrichment analysis network for biological function

**Figure 5:**
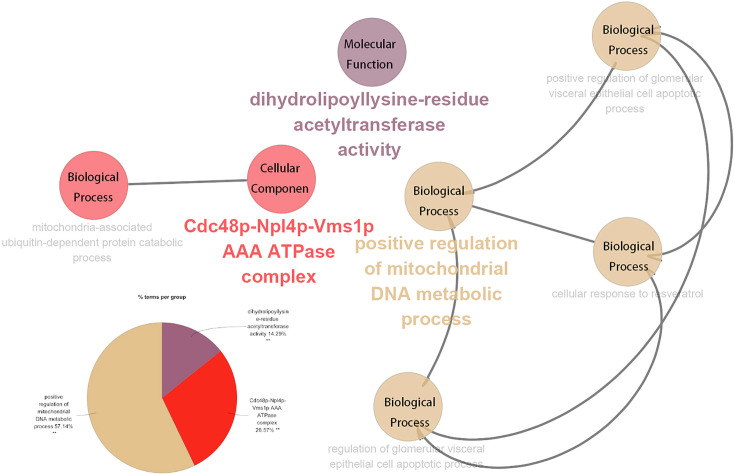
Analysis of biological function Interaction network diagram of biological function analysis of the seven genes that constitute the glycolysis model. The modules represented by specific colors are displayed in the pie chart (*P<0.05, **P<0.01).

Using the Cytoscape 3.7.1 software plug-in ClueGO, a biological function network of the seven glycolytic genes described above was constructed. The results indicate that the mitochondria-associated ubiquitin-dependent protein catabolic process interacts with the Cdc48p-Npl4p-Vms1p AAA ATPase complex; the cellular response to resveratrol, positive regulation of the mitochondrial DNA metabolic process, the regulation of the glomerular visceral epithelial cell apoptotic process, and positive regulation of the glomerular visceral epithelial cells apoptotic process jointly comprised an interactive network. The positive regulation of glomerular visceral epithelial cell apoptotic process accounted for the highest proportion of terms, accounting for 57.14%, with the Cdc48p-Npl4p-Vms1p AAA ATPase complex accounting for 28.57%. Dihydrolipoyllysine-residue acetyltransferase activity accounted for 14.29% (Figure 5).

### Construction of risk score related models of the seven glycolysis-related genes

The risk scores of the related genes were obtained using the formula: ∑ (coefficients of gene *n* × expression of gene *n*), and a relevant visualization model was constructed using R.

**Figure 6:**
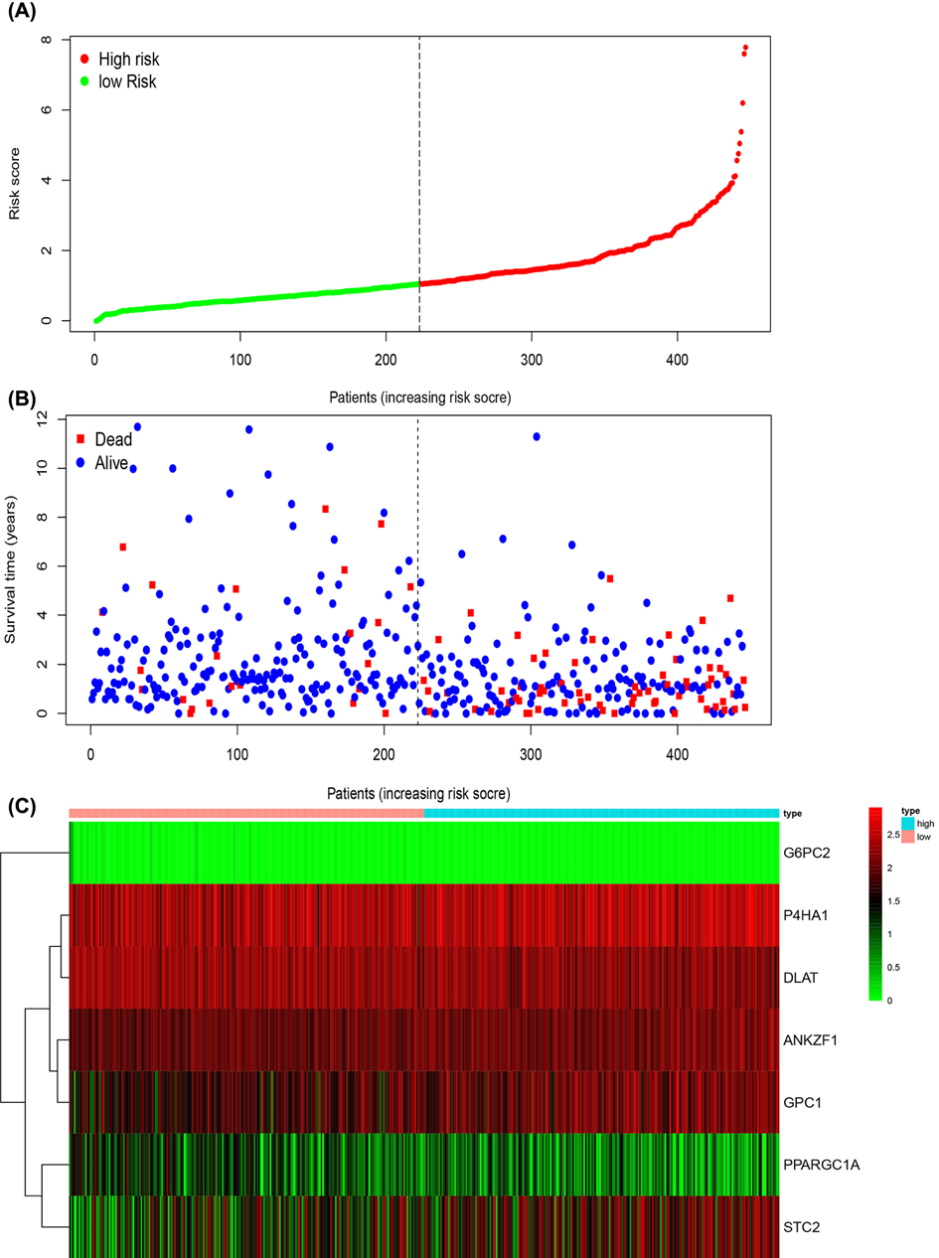
The seven‐mRNA signature associated with risk scores predicts overall survival in patients with colon adenocarcinoma (**A**) mRNA risk score distribution in each patient. (**B**) Survival in days of colon adenocarcinoma patients in ascending order of risk scores. (**C**) Heatmap of the expression profile of the seven genes.

The risk scores were stratified into two groups (Figure 6A), a low-risk group to the left of the dashed line, and a high-risk group to the right. As risk scores increased, the death of the patients was correspondingly shortened (Figure 6B), verifying that higher risk scores led to poorer patient survival. Finally, genes conferring protection (Figure 6C), such as PPARGC1A, DLAT, and G6PC2 appear to be relatively less expressed in the high-risk group, while the expression of those that confer risk, such as P4HA1, STC2, ANKZF1, and GPC1, was the converse.

### Relationship between clinical data and prognosis of colon adenocarcinoma

**Figure 7:**
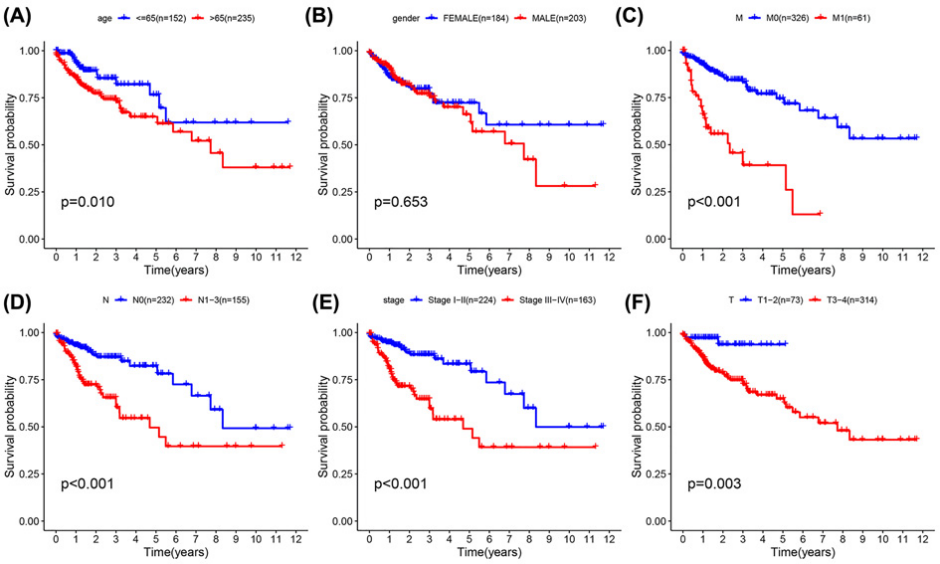
Kaplan–Meier survival analysis for COAD patients in TCGA data set Kaplan–Meier survival analysis of clinical features and survival rate. Clinical features included (**A**) (age), (**B**) (grade), (**C**) (M), (**D**) (N), (**E**) (stage), and (**F**) (T).

**Figure 8:**
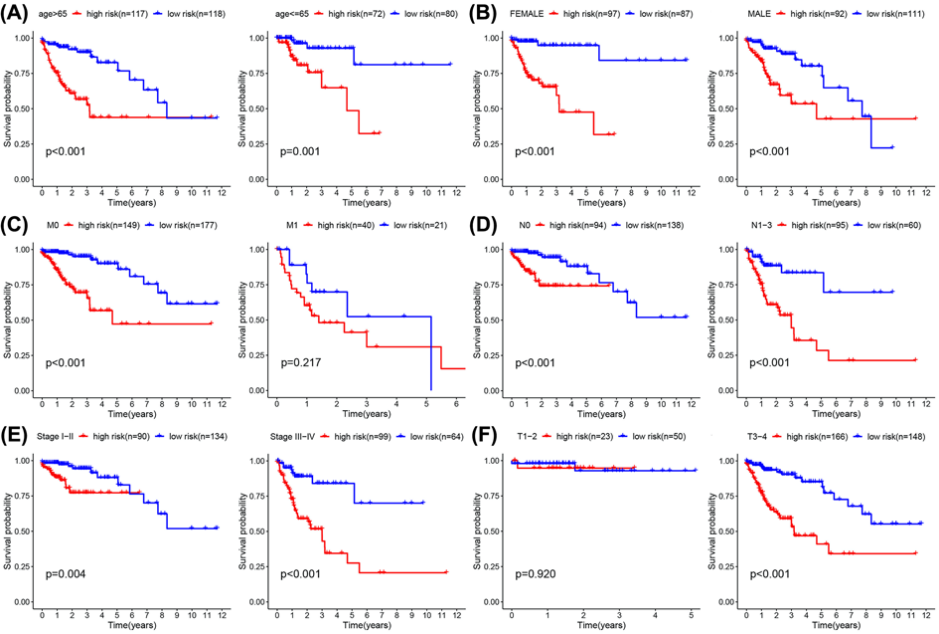
K-M survival analysis for COAD patients in TCGA data set K-M curves for prognosis of risk scores for the patients categorized by clinical feature. (**A**) (age), (**B**) (gender), (**C**) (M), (**D**) (N), (**E**) (stage), (**F**) (T).

As displayed in Table 4, the clinical data for a number of patients was unknown, and so the associated gene expression data were deleted. The data for the remaining 387 patients were analyzed and correlations between clinical data and prognosis for survival calculated, from which K-M curves were plotted. Furthermore, the results indicate that age, T, N, and M categories, and staging, are clinically and statistically significant for prognosis, and also accord with well-known patterns of COAD progression. Although gender is not statistically significant, we found that after 3 years of survival, men fare significantly worse than women, and we believe that this may signify particular clinical significance. Finally, the correlation between clinical data and risk scores was directly analyzed (Figure 7). Although for M1 patients, where *P*>0.05, the trend suggests that the survival rate of the high-risk group was still lower than that of the low-risk group, so it is judged that it still has clinical significance. In T patients, only those that are T3-4 do the high and low-risk groupings have clinical and statistical significance. Figure 8.

**Table 4:** Clinical data of patients with colon adenocarcinoma in the present study

Clinical information	Number	%	Dead number
**Age**			
≥65	283	0.63	65
<65	169	0.37	23
**Gender**			
Male	238	0.53	51
Female	214	0.47	37
**Stage**			
Stage I-II	254	0.56	30
Stage III-IV	187	0.41	53
Unknown	11	0.02	5
**Disease type**			
Cystic, Mucinous and Serous Neoplasms	64	0.14	15
Adenomas and Adenocarcinomas	379	0.84	71
Unknown	9	0.02	2
**T**			
T1-2	87	0.193	7
T3-4	364	0.805	81
Unknown	1	0.002	
**N**			
N0	269	0.60	34
N1-3	183	0.40	54
**M**			
M0	334	0.74	46
M1	62	0.14	27
Unknown	56	0.12	15

### Relationship between the glycolysis risk scores model and cells in the tumor microenvironment

**Figure 9:**
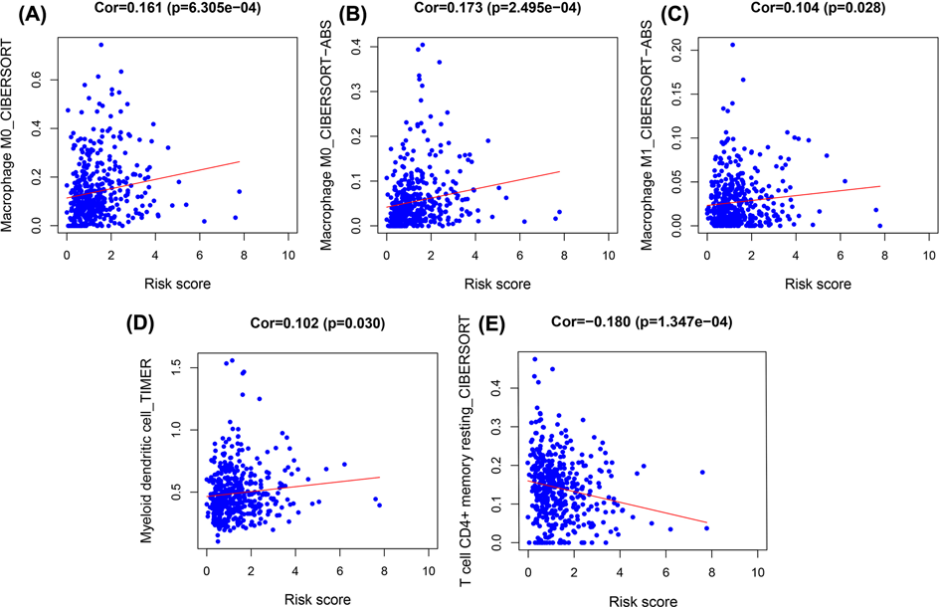
Trend between risk score of the glycolysis model and the number of cells that comprise the microenvironment (**A**)(Macrophage M0_CIBERSORT), (**B**) (Macrophage M0_CIBERSORT-ABS), (**C**) (Macrophage M1_CIBERSORT-ABS), (**D**) (Myeloid dendritic cell_TIMER), (**E**) (T-cell CD4+ memory resting_CIBERSORT).

**Figure 10:**
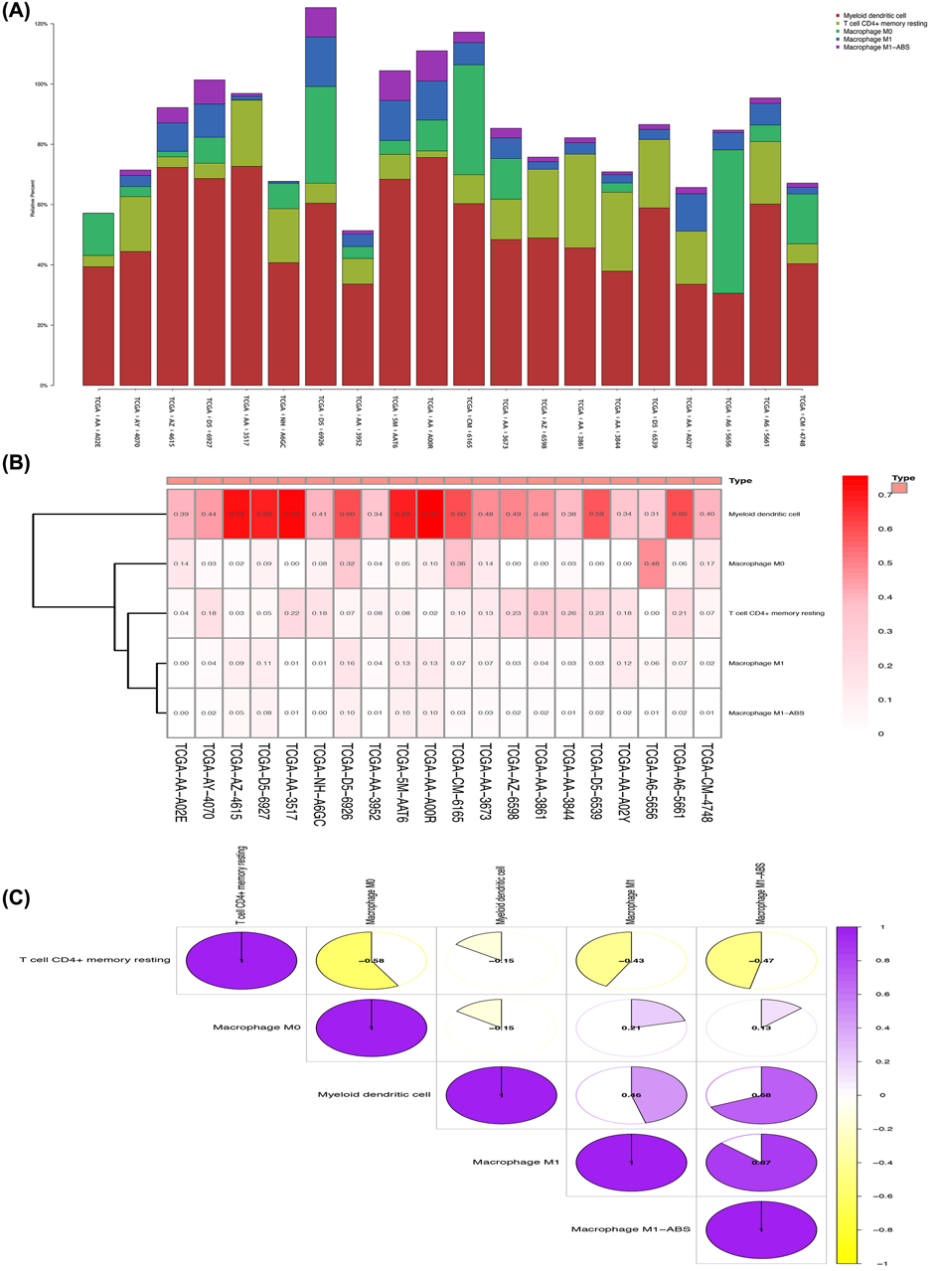
Selection of the 10 groups (left) of samples with the highest risk scores and 10 groups (right) of samples with the lowest risk scores in correlation analysis (**A**) The difference in cell numbers in the 20 samples. (**B**) Heat map of immune cell expression of the 20 sets of samples. (**C**) Immune cell correlation matrix, positive correlations displayed in purple, and negative correlations in gold.

Correlation analysis was conducted between the glycolysis risk scores model and cells in the tumor microenvironment using the TIMER2.0 database, finding that the number of macrophages, myeloid dendritic cells, and resting CD4+ memory T-cells changed significantly with differences in risk score (Figure 9). The number of myeloid dendritic cells was greatest in the selected samples, and macrophages most strongly correlated with myeloid dendritic cells (Figure 10) [14,15].

## Discussion

Research and clinical studies have shown that simple clinical traits are not sufficient to predict the development of tumors. Therefore, additional gene targets and detection markers are required to diagnose and predict tumor outcomes. According to research performed by Li et al., the markers for tumors in COAD are CEA and CA19-9 [16]. These two tumor markers allow additional indicators for identification and would allow additional speculation about COAD in clinical cases. However, current tumor markers are not particularly accurate for detecting the progression of COAD. Individual genes or markers are susceptible to a variety of factors and the influence of related gene regulation. It is difficult to accurately determine the information required to predict patient prognosis. Thus, researchers have hypothesized that multiple genes could be used to reduce interference and improve diagnosis. In terms of predicting tumor-related prognosis, the accuracy of statistical models and the reduction in interference are superior to single genes or markers [17]. As high-throughput sequencing technology has developed and databases are increasingly developed, big data can be used to explore statistical models for prediction of tumor prognosis [5]. Expression data from a large number of genes in each sample can be conveniently processed using big data analysis [18]. Research databases can provide related gene expression data to build models to predict the prognosis of specific tumors [19].

The principal of the Warburg effect is for the energy supply of tumor cells to change from oxidative phosphorylation to glycolysis. This change is regulated by multiple factors, including the tumor microenvironment and genetic changes [10]. The one gene-one enzyme hypothesis suggests that greater numbers of glycolysis-related genes associated with prognosis of COAD would result in greater numbers of genes that control the glycolytic process, providing additional targets for its clinical diagnosis and treatment in the future [20]. To enrich the number of glycolysis-related genes, 5 glycolysis-related datasets were queried using GSEA to identify glycolysis-related genes for study. In total, 326 glycolysis genes were identified. The expression of the glycolytic genes was compared in tumor tissues with tissue that was healthy and analyzed using a Wilcoxon test. Finally, 253 glycolytic genes differentially-expressed in tumor and normal tissue were obtained [21]. By linking clinical data with the expression of genes in the samples using Cox regression analysis, 7 genes were identified that constitute glycolysis-related genes for prediction of survival. The model can predict the difference in survival between high and low-risk score groups, with a sensitivity in the ROC model that is relatively high, suggesting that the risk score model that was constructed was relatively reliable. There are two principal forms of COAD (Adenomas and Adenocarcinomas; and Cystic, Mucinous and Serous Neoplasms). The difference in risk scores between the two disease types was not statistically different (*P*=0.6471), suggesting that the glycolysis risk scores model was applicable.

The co-expression heat map demonstrates that PPARGC1A has the greatest positive correlation with DLAT. When considering Cox regression analysis of risk scores against clinically relevant traits, staging data was included rather than T, N, M data due to the stage categories incorporating T, N, and M. The results show that for risk scores, *P*<0.001 and HR> 1, statistically and clinically significant differences existed. In terms of gene mutation, PPARGC1A contains the highest proportion of mutations, accounting for 4% of the total. Gene expression data indicated that the expression of genes was consistent with the results of the multivariate Cox regression analysis. The expression of risk factor genes was higher in tumors, and the expression of protective factor genes higher in normal colon tissue. The ClueGo plug-in was used to explore the biological function of the 7 genes identified in the study, finding that 57.14% of biological functions were enriched in the positive regulation of the mitochondrial DNA metabolic process, 28.57% of the biological functions were enriched in the Cdc48p-Npl4p-Vms1p AAA ATPase complex, and 14.29% in dihydrolipoyllysine-residue acetyltransferase activity. Detailed information of the 7 genes from which the risk scores model was constructed is shown in Table 5. Combined with this data, the overall trend suggests that increased patient risk scores are correlated with decreased survival time. The survival curve explores the relationship between risk scores and clinical data. Some of the clinical data that are routinely considered to exacerbate tumor were also consistent with the risk score trend of the model, further verifying its reliability.

**Table 5:** Detailed information of the seven genes that build the risk scores model

Official Symbol	Official Full Name	Biological function
PPARGC1A	PPARG coactivator 1 alpha	The protein encoded by this gene is a transcriptional coactivator that regulates the genes involved in energy metabolism.
P4HA1	prolyl 4-hydroxylase subunit alpha 1	This gene encodes a component of prolyl 4-hydroxylase, a key enzyme in collagen synthesis composed of two identical alpha subunits and two beta subunits
STC2	stanniocalcin 2	This gene encodes a secreted, homodimeric glycoprotein that is expressed in a wide variety of tissues and may have autocrine or paracrine functions.
ANKZF1	ankyrin repeat and zinc finger peptidyl tRNA hydrolase 1	The expression of this gene in lymph nodes is higher than other tissues, and there is no detailed explanation of biological function.
DLAT	dihydrolipoamide S-acetyltransferase	This gene encodes component E2 of the multi-enzyme pyruvate dehydrogenase complex (PDC).
G6PC2	glucose-6-phosphatase catalytic subunit 2	This gene encodes an enzyme belonging to the glucose-6-phosphatase catalytic subunit family.
GPC1	glypican 1	Cell surface heparan sulfate proteoglycans are composed of a membrane-associated protein core substituted with a variable number of heparan sulfate chains.

Li et al. observed a direct correlation between PPARGC1A, zinc-finger transcription factor snail homolog 1 (SNAI1), and metastatic lung disease, which promotes the metastasis of lung cancer. It has been proposed that this molecule is considered a potential biomarker for lung cancer prognosis [22]. Cao et al. established that the P4HA1/HIF1+ feedback loop drives glycolysis and the malignant phenotype of pancreatic cancer, and that gene silencing of P4HA1 significantly inhibited the proliferation of pancreatic ductal adenocarcinoma cells [23]. Li et al. found that after STC2 was silenced, the survival capability, migration, and invasion of colorectal cancer cells declined significantly [24]. Research by Zhou et al. demonstrated that high ANKZF1 expression was associated with low overall survival of colon cancer by participating in angiogenesis and a number of cancer signaling pathways [25]. Goh et al. considered that DLAT is a subunit of the pyruvate dehydrogenase complex, and future mechanistic studies should elucidate the mode of action of DLAT in human gastric cancer, establishing DLAT as a viable drug target [26]. Boortz et al. demonstrated that the absence of G6pc2 limited the increase in fasting blood glucose and improved glucose tolerance [27]. Melo et al. demonstrated that GPC1(+) crExos can be utilized as a potential non-invasive diagnostic and screening marker for the detection of early stages of pancreatic cancer, thereby allowing the possibility of surgical treatment [28]. GPC1 significantly affects the growth of pancreatic cancer cells *in vivo* and significantly attenuates tumor angiogenesis and metastasis in athymic mice [29]. Although these genes lack detailed studies in the field of colon adenocarcinoma, we can conclude from published studies that they possess the capability to promote tumor progression. Further exploration of the biological function of genes revealed that PPARGC1A and G6PC2 are both enriched in “gluconeogenesis”. DLAT, PPARGC1A, and G6PC2 are collectively enriched in “glucose metabolic process” and “hexose metabolic process”. STC2 and PPARGC1A are both enriched in the “cellular response to hypoxia”. We found that their biological function is esentially related to glucose metabolism and hypoxia, and related functions such as glucose metabolism and hypoxia, as described earlier, can promote tumor progression. The co-expression heat map indicates that PPARGC1A has the greatest positive correlation with DLAT, and they together regulate the glucose and hexose metabolic processes [30]. However, detailed connections between these genes requires further exploration. A more detailed study of the mechanisms of these genes in colon adenocarcinoma is required.

Research suggests that glycolysis in tumors is closely related to the tumor microenvironment [7,8,31]. Glycolytic activity was previously correlated with active immune signatures in cancer, as highly glycolytic tumors present an immune-stimulatory tumor microenvironment, and even correlating with immune checkpoints such as PD-L1 expression in tumors [8]. The 7 genes that constitute the glycolysis risk scores model were studied and their relationship with the immune microenvironment further investigated. As mentioned above, the tumor microenvironment is correlated with the occurrence and development of tumors [6]. In additional research, we found that the number of myeloid dendritic cells and macrophages increased as risk scores increased, and both are constituent cells of the immune microenvironment. However, we also found that the number of resting CD4+ memory cells decreased with increasing risk score. These observations suggest that COAD achieves this effect through the immune escape mechanism. Twenty samples were selected, 10 with the highest risk scores and 10 samples with the lowest, and the numbers of related immune cells measured. It was found that the number of myeloid dendritic cells in the 10 samples with the highest risk score was relatively higher. Associated research indicates that myeloid dendritic cells are correlated with the occurrence and development of many types of tumor, which may possibly affect risk score [32,33]. The results of this research highlight that myeloid dendritic cells mainly results from lymphatic metastasis of COAD. Myeloid dendritic cells can eliminate inflammation by inhibiting the activity of macrophages, but their regulatory mechanism in colon cancer requires further study [34].

## Conclusions

A total of seven genes (PPARGC1A, DLAT, 6PC2, P4HA1, STC2, ANKZF1, and GPC1) were found and used to constitute the glycolysis risk scores model for COAD. The model was able to predict patient prognosis of survival in colon adenocarcinoma.

## Data Availability

The data used to support the findings of this study are included within the article.

## Abbreviations

COAD: colon adenocarcinoma
coef: correlation coefficient
HR: hazard ratio
K-M: Kaplan–Meier curve
M: metastasis stage
N: lymph node metastasis stage
ROC: receiver operating characteristic
T: stage of primary tumor
